# Case Report: Fatal Complications of BK Virus-Hemorrhagic Cystitis and Severe Cytokine Release Syndrome Following BK Virus-Specific T-Cells

**DOI:** 10.3389/fimmu.2021.801281

**Published:** 2021-12-17

**Authors:** Elizabeth M. Holland, Corina Gonzalez, Elliot Levy, Vladimir A. Valera, Heather Chalfin, Jacquelyn Klicka-Skeels, Bonnie Yates, David E. Kleiner, Colleen Hadigan, Hema Dave, Haneen Shalabi, Dennis D. Hickstein, Helen C. Su, Michael Grimley, Alexandra F. Freeman, Nirali N. Shah

**Affiliations:** ^1^ Pediatric Oncology Branch, Center for Cancer Research (CCR), National Cancer Institute (NCI), National Institutes of Health (NIH), Bethesda, MD, United States; ^2^ Immune Deficiency- Cellular Therapy Program, Center for Cancer Research (CCR), National Cancer Institute (NCI), National Institutes of Health (NIH), Bethesda, MD, United States; ^3^ Radiology and Imaging Sciences, NIH Clinical Center (CC), Bethesda, MD, United States; ^4^ Urologic Oncology Branch, Center for Cancer Research (CCR), National Cancer Institute (NCI), National Institutes of Health (NIH), Bethesda, MD, United States; ^5^ Pediatric Oncology, Children’s National Medical Center, Washington, DC, United States; ^6^ Laboratory of Pathology, National Cancer Institute (NCI), National Institutes of Health (NIH), Bethesda, MD, United States; ^7^ Pediatric Gastroenterology, NIH Clinical Center (CC), Bethesda, MD, United States; ^8^ Laboratory of Clinical Immunology and Microbiology, National Institutes of Allergy and Infectious Disease, NIH Clinical Center (CC), Bethesda, MD, United States; ^9^ Division of Bone Marrow Transplantation and Immune Deficiency, Cincinnati Children’s Hospital, Cincinnati, OH, United States

**Keywords:** DOCK8 immunodeficiency syndrome, HSCT = hematopoietic stem cell transplant, BK virus associated hemorrhagic cystitis, virus specific T-cells, cytokine release syndrome

## Abstract

BK virus (BKV)-hemorrhagic cystitis (HC) is a well-known and rarely fatal complication of hematopoietic stem cell transplantation (HSCT). Treatment for BKV-HC is limited, but virus-specific T-cells (VST) represent a promising therapeutic option feasible for use posttransplant. We report on the case of a 16-year-old male with dedicator of cytokinesis 8 (DOCK8) deficiency who underwent haploidentical HSCT complicated by severe BKV-HC, catastrophic renal hemorrhage, and VST-associated cytokine release syndrome (CRS). Gross hematuria refractory to multiple interventions began with initiation of posttransplant cyclophosphamide (PT/Cy). Complete left renal arterial embolization (day +43) was ultimately indicated to control intractable renal hemorrhage. Subsequent infusion of anti-BK VSTs was complicated by CRS and progressive multiorgan failure, with postmortem analysis confirming diagnosis of hepatic sinusoidal obstruction syndrome (SOS). This case illustrates opportunities for improvement in the management of severe BKV-HC posttransplant while highlighting rare and potentially life-threatening complications of BKV-HC and VST therapy.

## Introduction

Allogeneic hematopoietic stem cell transplantation (HSCT) represents the only curative therapy for reversal of the clinical and immunological phenotype of dedicator of cytokinesis 8 (DOCK8) deficiency (1–5), an autosomal recessive combined immunodeficiency characterized by eczematous dermatitis, sinopulmonary infections, allergy, susceptibility to DNA viral infections, elevated serum IgE, and cancer predisposition (6, 7). Risk of post-HSCT viral reactivation and complications thereof remains elevated in this disease (5) and may be especially high in patients undergoing HSCT conditioning with posttransplant cyclophosphamide (PT/Cy). BK virus (BKV)-hemorrhagic cystitis (HC) can contribute to significant morbidity following HSCT but is seldom associated with fatal complications. Treatment options for BKV-HC are limited. While complications of BKV-HC are rarely life-threatening, we report on and discuss the management of a patient with BK viremia whose post-HSCT course was uniquely complicated by severe BKV-HC with combined renal hemorrhage and significant cytokine release syndrome (CRS) following treatment with BK-targeted virus specific T-cells (VSTs).

## Case

**Figure 1:**
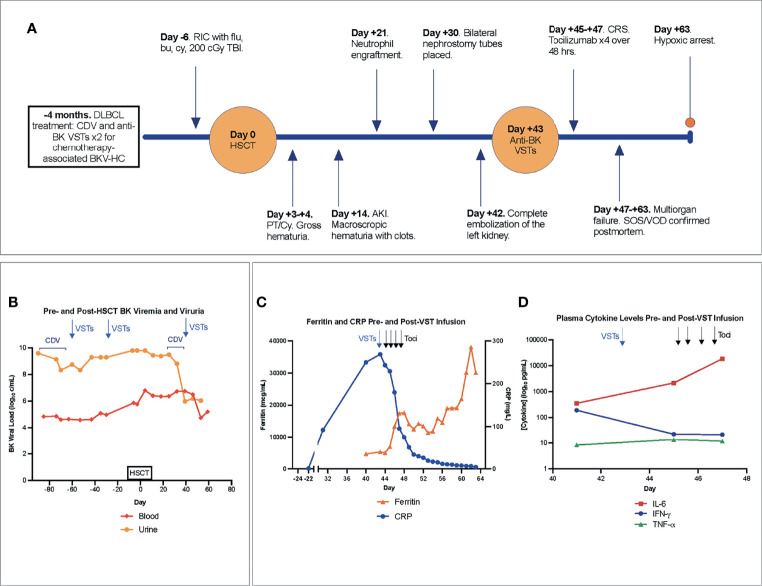
**(A)** Timeline of major events pre- and post-HSCT. **(B)** BK viremia and viruria with therapeutic interventions denoted in the pre- and post-transplant period. **(C)** Blood ferritin, CRP, and **(D)** plasma cytokine levels pre- and post-onset of CRS, with arrows indicating VST infusion (blue) and delivery of each of 4 total doses of tocilizumab (black). Notably, the first dose of tocilizumab was administered after cytokine levels were drawn on day +45. AKI, acute kidney injury; Bu, busulfan; CDV, cidofovir; CRP, C-reactive protein; CRS, cytokine release syndrome; Cy, cyclophosphamide; DLBCL, Diffuse large B-cell lymphoma; Flu, fludarabine; HSCT, hematopoietic stem cell transplantation; IFN-γ, interferon gamma; IL-6, interleukin-6; VST, quadrivalent anti-CMV, -EBV, -ADV, and -BK virus specific T-cells; PT/Cy, post-transplant cyclophosphamide; RIC, reduced intensity conditioning; SOS, sinusoidal obstructive syndrome; TBI, total-body irradiation; Toci, tocilizumab; TNF-α, Tumor Necrosis Factor alpha; VOD, veno-occlusive disease.

A 16-year-old male with DOCK8 deficiency (homozygous for *DOCK8* variant NM_203447.3:c.4153+1G>A) enrolled on an IRB-approved National Cancer Institute HSCT trial for patients with DOCK8 (NCT01176006). His disease, diagnosed at age 8 years, manifested with recurrent sinopulmonary infections, chronic molluscum contagiosum, and eczematous dermatitis. Recent complications included diagnosis of Diffuse Large B-Cell Lymphoma (DLBCL) 4 months pre-HSCT. Treatment with rituximab (4 doses) and LMB regimen (8, 9) (2 cycles), including vincristine, doxorubicin, corticosteroids, methotrexate and with cumulative cyclophosphamide dose of 3300 mg/m^2^ as per ANHL1131, Group B (R-COPADM), was complicated by chemotherapy-associated grade III BKV-HC managed with cidofovir and 2 infusions of third-party donor-derived quadrivalent anti-cytomegalovirus (CMV), -Epstein-Barr virus (EBV), -adenovirus (ADV), and -BK virus specific T-cells (VSTs) at 5x10^7^ cells/m^2^ (NCT02532452) (10). VSTs were well tolerated without any infusion reaction. HC symptoms subsequently resolved while asymptomatic BK viremia persisted. Achieving a complete remission, he proceeded to HSCT with his father as the haploidentical bone marrow donor. Reduced intensity (RIC) conditioning was comprised of fludarabine 30 mg/m^2^ x 5 days (days -6 to -2), busulfan dosed with target area under the curve (AUC) of 3600-4000 uM.min/day x 3 days (days -4 to -2), cyclophosphamide 14.5 mg/kg x 2 days (days -6 and -5), and low-dose total body irradiation (TBI, 200 cGy) on day -1. Graft-versus-host disease (GVHD) prophylaxis was comprised of post-transplant cyclophosphamide (PT/Cy) on days +3 and + 4 along with mycophenolate mofetil (MMF) x 30 days and tacrolimus x 6 months, both of which started on day +5 (11). Mild cystitis without hematuria developed on HSCT day 0. BK viremia simultaneously increased from 588,844 copies/mL (3 days pre-HSCT) to 6,456,542 copies/mL on day +4 (**Figure 1A**).

His immediate post-HSCT course was complicated by gross hematuria and painful bladder spasms with PT/Cy (50 mg/kg) on days +3 and +4. Grade IV gross hematuria, managed with daily blood product transfusions, persisted alongside worsening acute kidney injury (AKI, day +14) (**Figure 1A**). Continued transfusion dependence led to significant fluid retention and 11 kg weight gain over 11 days. Daily platelet infusions were used to maintain platelet count ≥ 30 K/mcL, and platelet engraftment ≥ 50 K/mcL was not attained. Neutrophil engraftment was achieved at day +21; chimerism studies demonstrated 100% donor-derived cells without evidence of GVHD.

**Figure 2:**
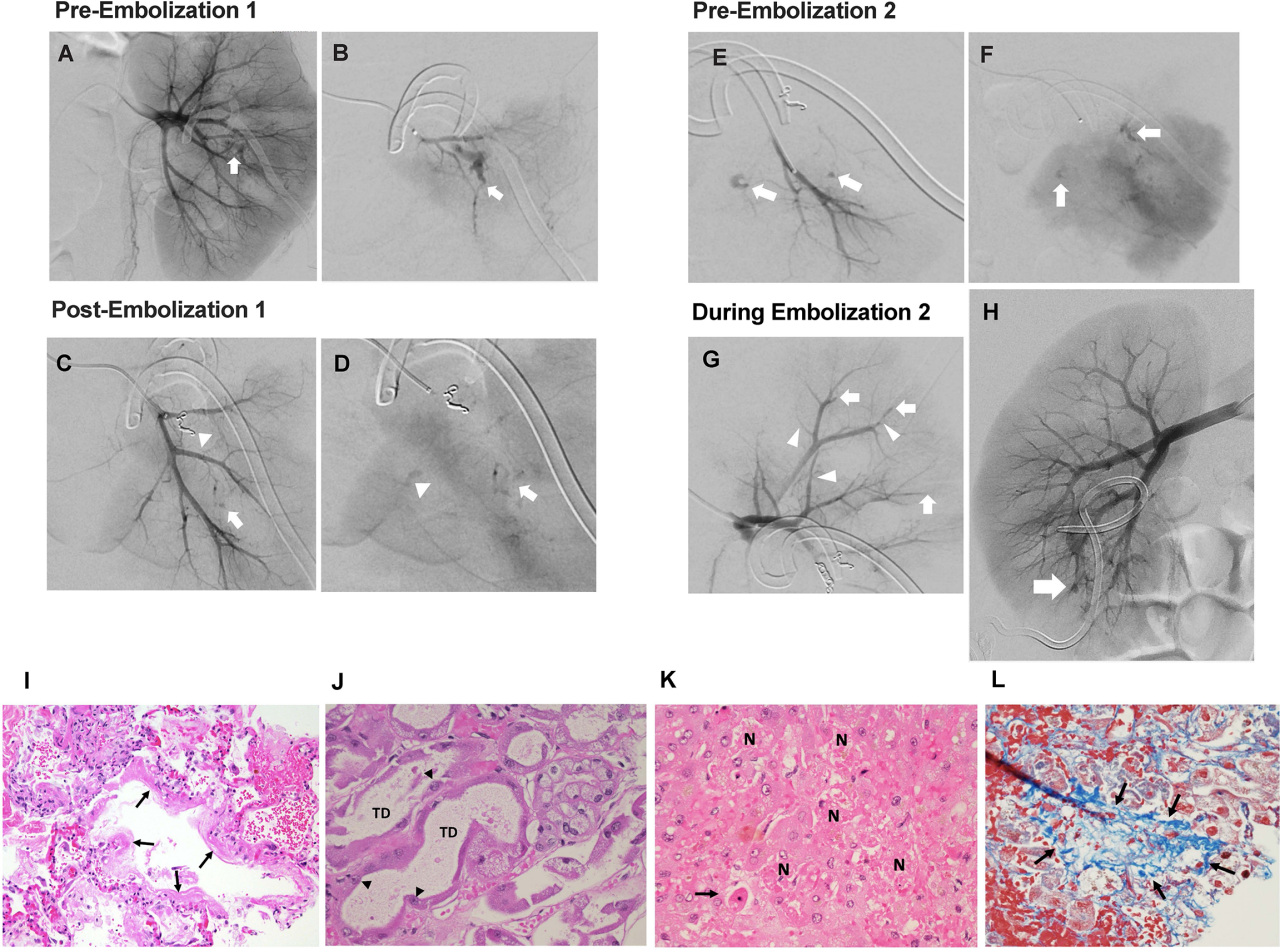
Interventional radiology findings. **(A)** Selective left renal arteriogram demonstrating active arterial bleeding in the midpole area (white arrow). **(B)** Superselective left renal arteriogram confirming active bleeding source (white arrow). **(C)** Superselective left renal arteriogram showing coil occlusion of arterial bleeding source (white arrowhead), as well as suspicious second bleeding source (white arrow). **(D)** Superselective left renal arteriogram (parenchymal phase) showing slower extravasation from same location as image C (white arrow), as well as third potential bleeding source (white arrowhead). **(E)** Superselective left renal arteriogram performed 72 hours after embolization redemonstrating bleeding sources (white arrows) in early arterial phase and delayed **(F)** images. **(G)** Superselective left upper pole renal arteriogram performed during second embolization showing distal vessel narrowing (white arrowheads) and occlusions (white arrows). **(H)** Right renal arteriogram performed during second left renal embolization showing a lower pole arteriovenous fistula (white arrow). Pathology findings. **(I)** Exudative phase diffuse alveolar damage with hyaline membrane formation (arrows) (H&E, 200x). **(J)** Acute tubular injury with tubular dilation (TD) and reactive atypia of surviving tubular epithelial cells (arrowheads) (H&E, 400x). **(K)** Hepatic necrosis (N) and apoptosis (arrow), (H&E, 400x). **(L)** Occluded hepatic vein filled with loose blue-stained collagen (arrows) (Masson trichrome, 400x).

Progressive hydronephrosis and hydroureter prompted Foley catheter and bilateral nephrostomy tube placement by day +30. Gross hematuria from the left nephrostomy tube occasioned left renal arteriography which revealed active bleeding unrelated to the nephrostomy at multiple sites and features suggestive of vasculitis. Coil embolization performed on two separate occasions (days +35 and +38) provided only transient stabilization. Ongoing bleeding and transfusion needs were accompanied by increasing total and direct hyperbilirubinemia (2.9 mg/dL and 2.7 mg/dL, respectively (day +43)). Complete left renal artery embolization on day +42, followed by initiation of intravenous cidofovir (**Figures 2A–H**), stabilized the patient for transport to receive an additional infusion of third-party VSTs (day +43) produced using the same donor as his second pre-HSCT infusion (NCT02532452). BK viremia measured 5,754,399 copies/mL at third infusion, nearly a full log increase from viral load with pre-transplant VSTs (**Figure 1B**).

Two days after VST infusion (day +45), onset of cytokine release syndrome (CRS) was evidenced by fever, hypotension, worsening lung opacities, and bilateral pleural effusions. Inflammatory markers indicative of CRS were also elevated. CRP peaked at 268.7 mg/L on day +43 following VST infusion. Interestingly, CRP had been rising in the days leading up to VST infusion, potentially in the context of significant bleeding and interventional procedures. A rapid rise in ferritin was seen from 4,641 mcg/mL pre-VSTs (day +40) to 5,313 mcg/mL post-infusion (day +45) and 17,456 mcg/mL on day +47 (**Figure 1C**). Plasma IL-6 rose from 360 pg/mL pre-infusion (day +41) to 2,182 pg/mL on day +45 with CRS onset and prior to initiation of the IL-6R (receptor) blocker tocilizumab (**Figure 1D**). Concurrent blood, urine, and viral testing remained negative for any signs of new infection. Aggressive fluid resuscitation, bilateral chest tube placement, vasopressor support, continuous renal replacement therapy (CRRT), and 4 doses of tocilizumab (8 mg/kg) were given over 48 hours. Following fluid resuscitation for CRS, liver studies (day +47) demonstrated worsening hyperbilirubinemia. Liver ultrasound showed hepatosplenomegaly and sluggish flow through the main portal vein, raising concern for late-onset sinusoidal obstruction syndrome (SOS)/veno-occlusive disease (VOD). Given prior life-threatening hemorrhage, defibrotide was contraindicated. Multiorgan failure and worsening coagulopathy led to hypoxic arrest on day +63. Autopsy was declined but limited postmortem single core liver, kidney, and lung biopsies demonstrated hepatic SOS/VOD with zone 3 hemorrhagic necrosis, acute renal tubular injury, and early pulmonary exudative phase diffuse alveolar damage (**Figures 2I–L**). Limited tissue SV40 immunostain for polyomavirus was negative at all 3 sites.

## Discussion

Complications of post-transplantation viral reactivation remain a significant challenge in patients with DOCK8 deficiency due to the underlying immunodeficiency associated with DOCK8 mutation. We highlight unique challenges of managing BKV-HC with hemorrhagic nephropathy and describe potential complications of anti-BK VST therapy.

Inclusion of PT/Cy in HSCT conditioning has been associated with a particularly high degree of HC (10, 12). PT/Cy may exacerbate bleeding predisposition by inflicting substantial damage to the urothelium of a robustly immunosuppressed and immunocompromised host. In our patient, BKV-associated bleeding may have been suggestive of a propensity for endothelial damage already underway, with late-onset VOD/SOS developing consequently.

It is unclear if our patient’s bleeding complications were associated with high BK viremia post-HSCT, an unusual manifestation of BKV nephropathy (13, 14), or were exacerbated by an underlying immunodeficiency-related vasculopathy. Postmortem analysis did not show evidence of renal polyomavirus, but limited sampling not representative of cross-sectional involvement or false negativity may have precluded the ability to detect BKV (15).

Renal arteriography performed to control left renal hemorrhage demonstrated findings suggestive of vasculopathy including irregular luminal contour, peripheral branch occlusions, and arterial extravasation. DOCK8 deficiency has been associated with vasculitis leading to aortic aneurysm, calcification, or stroke (7, 16–18). Vascular changes noted at arteriography showed distribution at the distal arterial branches and arcuate arteries in the left kidney, which more closely corresponds with vessels affected by immune complex small vessel vasculopathies. Although vasculitis could be associated with hematologic malignancy (DLBCL), viral infection, or DOCK8 mutation, specific attribution is speculative at best.

Management of BK viremia and BKV-HC with current supportive care measures, including cidofovir, has shown only suboptimal benefit (19, 20). Given limited options for BKV-HC treatment, anti-BK VSTs address a critical vulnerability in patients with poor or absent T-cell mediated immunity and are feasible for posttransplant use. Using HSCT-donor derived VSTs, Nelson et al. found that 87% of patients with BK viremia and/or HC (n=38) responded to ≥1 anti-BK VST infusions; 58% completely cleared BKV and demonstrated resolution of HC symptoms ≤ grade II (10). Olson et al. describe comparable outcomes with HLA-matched third-party anti-BK VSTs (21). Infusion-related adverse events were rare with HSCT-donor derived or third-party VSTs (10, 21).

Toxicity concerns with allogeneic adoptive T-cell transfer include GVHD and CRS (22). One study of donor-derived and third-party “off-the-shelf” VSTs reported mild GVHD in 11% of patients with primary immunodeficiencies, with all cases suspected to be transplant-related rather than VST induced (23). CRS has been recorded in ≤ 2% of patients receiving VSTs (24). Our patient, however, developed CRS requiring significant support after infusion with the same VST product he previously tolerated. High viral load and disseminated disease have been suggested to increase risk of developing CRS post-VST infusion (25). Interestingly, in the days preceding post-HSCT VST infusion, our patient had a rising CRP. This was potentially associated with life-threatening hematuria and repeated renal interventions, as no obvious alternative infectious source was identified. We postulate that this pre-existing inflammatory milieu in the context of high BK viremia and donor chimerism (100%), with more reactive lymphocytes than in the pre-HSCT setting, likely all converged and collectively contributed to this severe degree of CRS.

BK viremia and BKV-HC can cause life-threatening complications following HSCT, particularly in those with immunodeficiency. Early HSCT consideration may improve outcomes in patients with DOCK8 deficiency, especially if HSCT can be performed prior to the development of comorbidities associated with the underlying disease which may complicate the transplant course. While proceeding to HSCT with active viremia and sequelae thereof is challenging, HSCT may also be the only path forward in those unable to fully clear their viral disease without immune reconstitution. Thus, management of underlying comorbidities should be optimized as feasible. The use of VSTs in the pre-HSCT setting in our case served to optimize control of BKV and associated BKV-HC prior to HSCT. Use of PT/Cy also requires careful monitoring, particularly with inclusion of cyclophosphamide in HSCT conditioning and cumulative HC risk. This case raises awareness of severe complications of BKV-HC. Further study of optimal management strategies of viral disease and severe BKV-HC posttransplant is needed.

## Data Availability Statement

The original contributions presented in the study are included in the article/supplementary material. Further inquiries can be directed to Nirali.Shah@nih.gov.

## Ethics Statement

The studies involving human participants were reviewed and approved by National Cancer Institute, Institutional Review Board. Written informed consent to participate in this study was provided by the participants’ legal guardian/next of kin.

## Author Contributions

EMH and NNS wrote the first version of the manuscript. CG, EL, VAV, HC, JK-S, BY, CH, HD, HS, DDH, HCS, MG, AFF, NNS provided direct patient care and/or guidance on management. DEK provided pathology review. All authors contributed to the final version of the manuscript.

## Author Disclaimer

The content of this publication does not necessarily reflect the views of policies of the Department of Health and Human Services, nor does mention of trade names, commercial products, or organizations imply endorsement by the U.S. Government.

## Conflict of Interest

The authors declare that the research was conducted in the absence of any commercial or financial relationships that could be construed as a potential conflict of interest.

## Publisher’s Note

All claims expressed in this article are solely those of the authors and do not necessarily represent those of their affiliated organizations, or those of the publisher, the editors and the reviewers. Any product that may be evaluated in this article, or claim that may be made by its manufacturer, is not guaranteed or endorsed by the publisher.

## Abbreviations

ADV, adenovirus; AKI, acute kidney injury; BKV, BK virus; CMV, cytomegalovirus; CRP, C-reactive protein; CRRT, continuous renal replacement therapy; CRS, cytokine release syndrome; DLBCL, Diffuse large B-cell lymphoma; DOCK8, dedicator of cytokinesis 8; EBV, Epstein-Barr Virus; GVHD, graft-versus-host disease; HC, hemorrhagic cystitis; HLA, human leukocyte antigen; HSCT, hematopoietic stem cell transplantation; LMB, Lymphomes Malins B therapy; PT/Cy, posttransplant cyclophosphamide; RIC, reduced intensity conditioning; SOS, sinusoidal obstruction syndrome; TBI, total body irradiation; VOD, veno-occlusive disease; VST, virus specific T-cell.
